# The Hybrid Genome of a New Goldfish-Like Fish Lineage Provides Insights Into the Origin of the Goldfish

**DOI:** 10.3389/fgene.2020.00122

**Published:** 2020-03-03

**Authors:** Yude Wang, Huifang Tan, Minghe Zhang, Rurong Zhao, Shi Wang, Qinbo Qin, Jing Wang, Chun Zhang, Min Tao, Ming Ma, Bo Chen, Shaojun Liu

**Affiliations:** ^1^ State Key Laboratory of Developmental Biology of Freshwater Fish, College of Life Sciences, Hunan Normal University, Changsha, China; ^2^ College of Chemistry and Chemical Engineering, Hunan Normal University, Changsha, China

**Keywords:** chimeric genes, full-length transcriptomes, goldfish-like homodiploid fish, gene structure, resequencing

## Abstract

Distant hybridization leads to obvious changes in genotypes and phenotypes, giving rise to species with novel capabilities. However, the fusion of distinct genomes also polymerizes the DNA or gene variations that occur during the course of evolution. Knowledge of the early stages of post-hybridization evolution is particularly important. Here, we investigated the full-length (FL) transcriptomes and the sequences resulting from the genome resequencing of the red crucian carp-like homodiploid fish (RCC-L) and goldfish-like homodiploid fish (GF-L) derived from the interspecific hybridization of koi carp (KOC) and blunt snout bream (BSB) to provide molecular evidence for the hybrid origin of the goldfish (GF). We compared the orthologous genes in the transcriptomes of RCC-L and GF-L with those of KOC and BSB. We also mapped the orthologous genes to the common carp (CC) and BSB genomes and classified them into eight gene patterns in three categories (chimaera, mutant, and biparental origin genes). The results showed that 48.20% and 46.50% of the genes were chimaera and that 3.70% and 8.30% of the genes were mutations of orthologous genes in RCC-L and GF-L, respectively. In RCC-L and GF-L, 63.70% and 68.20% of the genetic materials were from KOC, and 12.30% and 11.90% of the genetic materials were from BSB. The sequences from the genome resequencing of RCC-L and GF-L were mapped to the genome sequences of CC and BSB, revealing that the similarities of both RCC-L and GF-L to the CC genome (92.52%, 90.18%) were obviously higher than to the BSB genome (50.33%, 49.18%), supporting the suggestion that the genomes of both RCC-L and GF-L were mainly inherited from KOC but had some DNA fragments from BSB. Overall, our results provide molecular biological evidence for the hybrid origin of red crucian carp (RCC) and GF.

## Introduction

Goldfish (GF), which are popular pets among Chinese people, are cyprinid fish and are considered to be variants of crucian carp (Wang et al., 2014). However, the evolutionary origins of GF remain unknown. Some studies suggest that GF evolved from wild crucian carp (Chen et al., 2015). According to historical records, the GF derived from a wild silver-grey crucian carp that first mutated into a red-yellow gold crucian carp and was then domesticated during several periods (Watson et al., 1963; Fu and Shisan, 2016; Wang et al., 2018). However, this hypothesis lacks direct evidence. GF and wild crucian carp vary in shape and color. An obvious difference between GF and wild crucian carp is the distinctly split double tail of the GF. Generally, people think GF originated from crucian carp. Nevertheless, there is insufficient evidence to support the hypothesis.

**Figure 1:**
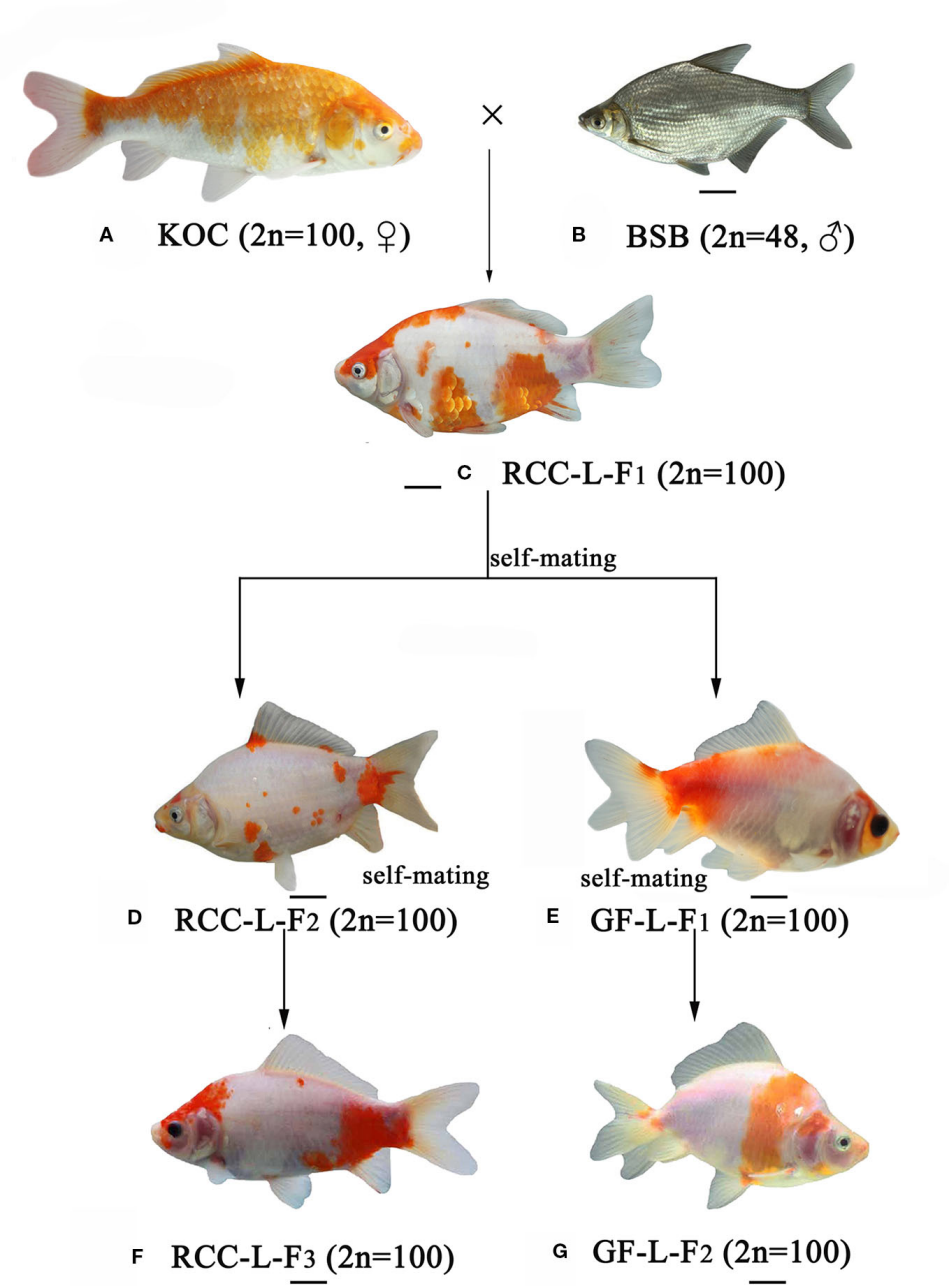
The formation of experimental ﬁsh hybrids. **(A)** KOC; **(B)** BSB; **(C)** RCC-L-F_1_; **(D)** RCC-L-F_2_; **(E)** GF-L-F_1_; **(F)** RCC-L-F_3_; **(G)** GF-L-F_2_, Bar = 3cm.

Distant hybridization is defined as interspecific hybridization, i.e., combining whole genomes from two distinct taxa, which may generate offspring with different phenotypes and genotypes (Biradar and Rayburn, 2016). Among all vertebrate classes, the distant hybridization of fish has been deliberately conducted between species, genera, subfamilies, and orders (https://trove.nla.gov.au/version/26056772). Most of the distant hybrid progenies are reported to be allotetraploid, autotetraploid, or allodiploid (Liu, 2010). However, few reports have focused on homoploid hybrids. Recently, we successfully obtained homodiploid hybrids of red crucian carp-like (RCC-L) homodiploid fish and goldfish-like (GF-L) homodiploid fish (Wang et al., 2018) (**Figure 1**). In terms of appearance, significant phenotypic differences were observed between GF-L, RCC-L, and their original parents (koi carp (KOC) and blunt snout bream (BSB)); for example, compared with KOC and BSB, RCC-L and GF-L have unique characteristics such as blue eyes, small heads and transparent bodies. Interestingly, the GF-L has beautiful twin tails. Based on the results of previous research comparing their 5S rDNA and simple sequence repeats (SSRs), we found that the GF-L and RCC-L were similar to natural GF and red crucian carp (RCC), respectively. However, we lack transcriptomic or genomic evidence concerning the molecular mechanisms underlying the RCC-L and GF-L hybrids.

Recently, a third-generation sequencing (TGS) platform was released that produces long but relatively low-quality reads up to 20 kb in length (Roberts et al., 2013). Although the quality of reads produced is low, this TGS platform is recognized as an outstanding technology for characterizing full-length (FL) transcripts, because long reads are of great importance in *de novo* genome and transcriptome assemblies (Au et al., 2013; Sharon et al., 2013; Huddleston et al., 2014; Xu et al., 2015). Long reads or FL cDNA sequences are the basis for structural genomics and functional genomics research. Whole-genome resequencing is used to sequence the individuals of a species with existing reference sequences, to analyze the differences at the individual or population level, and to filter out an abundance of variation information, such as single nucleotide polymorphisms (SNPs), insertions/deletions (Indels), structural variations (SVs), and copy number variations (CNVs), to obtain the genetic characteristics of the biological entity. The recently completed reference genomes of common carp (CC) and BSB allowed us to systematically analyze whole-genome resequencing data to determine the potential molecular characteristics of GF-L and RCC-L. Here, we generated whole-genome resequencing data for GF-L and RCC-L to evaluate their genetic relationship at the genome level, especially focus on determining the maternal components, the paternal components, and variant components of the offspring.

In this study, we characterized the gene structure changes in early generations of homodiploid lineages (RCC-L and GF-L) by comparing RCC-L and GF-L with their origin parents (KOC and BSB) using TGS and genome resequencing analyses. The results provided molecular biological evidence for the hybrid origin of the RCC and GF.

## Methods

### Materials

Two-year-old KOC, two-year-old BSB, one-year-old RCC-L, and one-year-old GF-L were cultured in the State Key Laboratory of Developmental Biology of Freshwater Fish, Hunan Normal University. These fish were anaesthetized with 100 mg/L MS-222 (Sigma-Aldrich, St. Louis, MO, USA) before dissection. Liver tissues were immediately frozen in liquid N_2_.

### RNA Preparation and cDNA Synthesis

Total RNA was extracted by grinding the collected tissue in TRIzol reagent (Sigma) on dry ice and further processed following the protocol provided by the manufacturer. To remove DNA, an aliquot of RNA was treated with DNase (TaKaRa, Dalian, Liaoning, China) and subsequently subjected to phenol/chloroform/isoamyl alcohol extraction using Phase Lock Gel Light tubes and ethanol precipitation. RNA precipitation was performed at -20°C for 0.5 h. First-strand cDNA synthesis was performed using the Clontech SMARTer PCR cDNA Synthesis Kit (Clontech 634925) following the manufacturer's instructions, with a total of 1 μg RNA for each cDNA synthesis reaction. The double-stranded cDNA product was subsequently amplified by PCR.

### PacBio cDNA Library Construction and TGS

The reverse transcription (RT) of 1 mg of total RNA per tissue per reaction was conducted in a separate PCR tube using the Clontech SMARTer PCR cDNA Synthesis Kit (Clontech 634925) and tissue-specific barcode oligo dT (with the PacBio 16-mer barcode sequence) (**Supplementary Table 1**) to generate barcoded FL cDNA. Two or three RT reactions per tissue were run in parallel. PCR optimization was applied to determine the optimal amplification cycle number for the downstream large-scale PCRs. A single primer (CDS Primer IIA from the Clontech SMARTer kit, namely, 5'-AAG CAG TGG TAT CAA CGC AGA GTA C-3') was used for all PCRs conducting RT, and the PCR products contained two fractions. Fraction one was purified twice using 1×AMPure^®^ PB beads, and fraction two was purified once using 1×AMPure^®^ PB beads. Then, the two fractions were mixed with equal molar quantities in a clean LoBind microcentrifuge tube for a non-size-selected single-molecule real-time (SMRT) bell library. Finally, annealing and binding were performed following the manufacturer's instructions (Pacific Biosciences). The non-size-selected Iso-Seq library was prepared and sequenced using Sequel DNA polymerase 2.0 on a Sequel platform with a 600 min run time (if the sequencing data size was over 10 GB, we used a 1,200 min run time).

Equimolar ratios of four cDNA libraries were pooled. A total of 6 µg of cDNA was subjected to fractionate on the basis of size using the Sage ELF system. Size fractions eluted from the run were subjected to the quality control and pooled in equimolar ratios for subsequent re-amplification to yield one library (0.5–6 kb). The mixed PCR products were purified using AMPure PB beads. One to five micrograms of purified amplicons were subjected to Iso-Seq SMRT Bell library preparation (https://pacibo.secure.force.com/SamplePrep). Three barcoded SMRT bell libraries (0.5–6 kb) were selected on size using the Sage Blue Pippin system to remove trace amounts of small inserts. A total of 47 SMRT cells were sequenced on the PacBio RS II platform using P6-C4 chemistry with 3–4 h run time.

### Quality Filtering and Error Correction of the PacBio Long Reads

First, we obtained the reads of insert (ROIs) after filtering the TGS subreads in the SMRT analysis software suite (http://www.pacificbiosciences.com) with default settings. Then, reads were examined by poly(A) signals and 5′ and 3′ adaptors, and FL and non-FL cDNA reads were recognized. Finally, an iterative clustering algorithm was used to identify consensus isotypes for error correction, and these consensus isotypes were further polished into high-quality isotypes.

### Predictions of ORFs, Novel Genes, and lncRNAs

To predict the open reading frames (ORFs) in transcripts, we used the package TransDecoder v2.01 (https://transdecoder.github.io/) to confirm putative coding sequences (CDSs). Predicted CDSs were searched and confirmed by BLASTX (E-value ≤ 1e-5) against three protein databases (non-redundant (NR), Swiss-Prot, and Kyoto Encyclopedia of Genes and Genomes (KEGG)). Those transcripts containing complete ORFs, 5′-untranslated regions (UTRs) and 3′-UTRs were regarded as FL transcripts. To ensure putative simple sequence repeats (SSRs) in our assembled sequences, the Microsatellite Identification (MISA) tool (http://pgrc.ipk-gatersleben.de/misa) was used. Only transcripts that were greater than or equal to 500 bp in size were applied to SSR detection. To confirm long non-coding RNAs (lncRNAs), we applied PLEK software (predictor of lncRNAs and mRNAs based on an improved k-mer scheme; https://sourceforge.net/projects/plek). LncRNA prediction was performed on reads that were compared with the genome. But LnRNAs reads without being annotated and predicted were considered as novel genes.

### Fusion Gene Identification

The FL transcripts in RCC-L and GF-L were BLAST searched to the genomic contigs in the CC and BSB genomes, respectively. The alignment hits between each given transcript and the best-matched contig were collected and filtered using the following parameters: the number of the same matched hits was greater than or equal to 25 and percentage of consistency with matched hits was greater than or equal to 90%. The mismatched or redundant alignment hits were further filtered, and the exon-intron structure of each transcript was confirmed using local scripts. Later, the numbers and lengths of exons and introns were statistically analyzed. To improve and validate our local scripts, we downloaded the datasets of genomic sequences of the CC and fitted our working pipeline to this well-characterized species.

### Evaluation of Ka/Ks in Putative Orthologous Gene Pairs

The orthoMCL algorithm with default settings was used to discern orthologous gene pairs between GF-L, RCC-L, KOC, and BSB. If a transcript in one species had multiple possible orthologues in the other species, the transcript was deleted. For each orthologous gene pair, sequence alignment was executed with the MUSCLE program (http://drive5.com/muscle; Edgar, 2004). Then, the non-synonymous and synonymous substitution rates (Ka and Ks) were evaluated using the YN00 program for phylogenetic analysis.

### Mapping and Identities Using Genome Resequencing

DNA libraries were constructed with an insert size of approximately 500 bp according to the manufacturer's instructions (Illumina, San Diego, CA, USA). Libraries were sequenced using the Illumina HiSeq 2500 platform along with paired-end 150 bp reads. A total of 135,935,765,390 (135.94 G) and 97,789,869,732 (97.79 G) clean reads were generated from RCC-L and GF-L, respectively, producing a minimum of 10-fold genomic coverage. Then, we deleted the pair-end reads containing greater than or equal to 5% unidentified nucleotides (N), adaptor sequences, and low-quality reads. The high-quality reads were BLAST searched against the CC genome and BSB genome by applying BWA software with default options (Li and Durbin, 2009). For the genome comparison of RCC-L and GF-L, the RCC-L and the GF-L genome sequences were modelled as sequencing data with 90× read depth and aligned to the CC, BSB, and RCC genomes (Xu et al., 2014; Liu et al., 2016; Liu et al., 2017).

### Identification of SNPs and Variations Using DNA Resequencing

The local alignment of indel sequences was executed on deduplicated reads by using Realigner Target Creator and Indel Realigner in the genome analysis tool. Multi-sample SNP genotyping was performed to identify SNPs by using the unified Genotyper in GATK. To reduce the false discovery rate, we performed stringent filtering based on the following criteria: QUAL < 30.0, QD < 2.0, MQ < 40.0, FS > 60.0, Haplotype Score >13.0, MQ RankSum < -12.5, and Read PosRankSum < -8.0. Break Dancer software was used to detect SVs in the RCC-L and GF-L genomes. There were five types of SVs detected in the genome: insertions (INS), deletions (DEL), inversions (INV), inter-chromosomal translocations (CTX), and duplications (DUP).

## Results

### SMRT-Based RNA Sequencing

To obtain representative FL transcriptomes for GF-L, RCC-L, KOC, and BSB, SMRT sequencing was performed using the Pacific RSII sequencing platform. For each organism, four liver tissue samples were collected from four different individuals and mixed, and the mixture was used for mRNA extraction. Three size-fractionated FL cDNA libraries (1–2 kb, 2–3 kb, and >3 kb) were constructed and subsequently sequenced in three SMRT cells. The percentages of high-quality FL non-chimeric PacBio reads were 15.45%, 51.41%, 44.90%, and 46.45% for GF-L, RCC-L, KOC, and BSB, respectively. The average lengths of FL non-chimeric PacBio reads were 1,968 bp, 1,893 bp, 1,893 bp, and 1,893 bp for GF-L, RCC-L, KOC, and BSB, respectively. The numbers of non-FL PacBio reads were 65,479 bp, 7,003 bp, 8,428 bp, and 7,832 bp for GF-L, RCC-L, KOC, and BSB, respectively (**Table 1**).

**Table 1:** Summary of the PacBio-based RNA sequencing in this study.

	GF-L	RCC-L	KOC	BSB
Number of non-full-length PacBio reads	65479	7003	8428	7832
Number of full-length non-chimeric PacBio reads	59274	91459	93736	114649
Average length of full-length non-chimeric PacBio reads (bp)	1968	1893	1893	1893
Number of non-redundant full-length transcripts after correction	55807	55837	61281	67473
High-quality full-length non-chimeric PacBio reads (bp)	8621	28705	27516	31341

### Prediction of New Genes and lncRNAs

**Figure 2:**
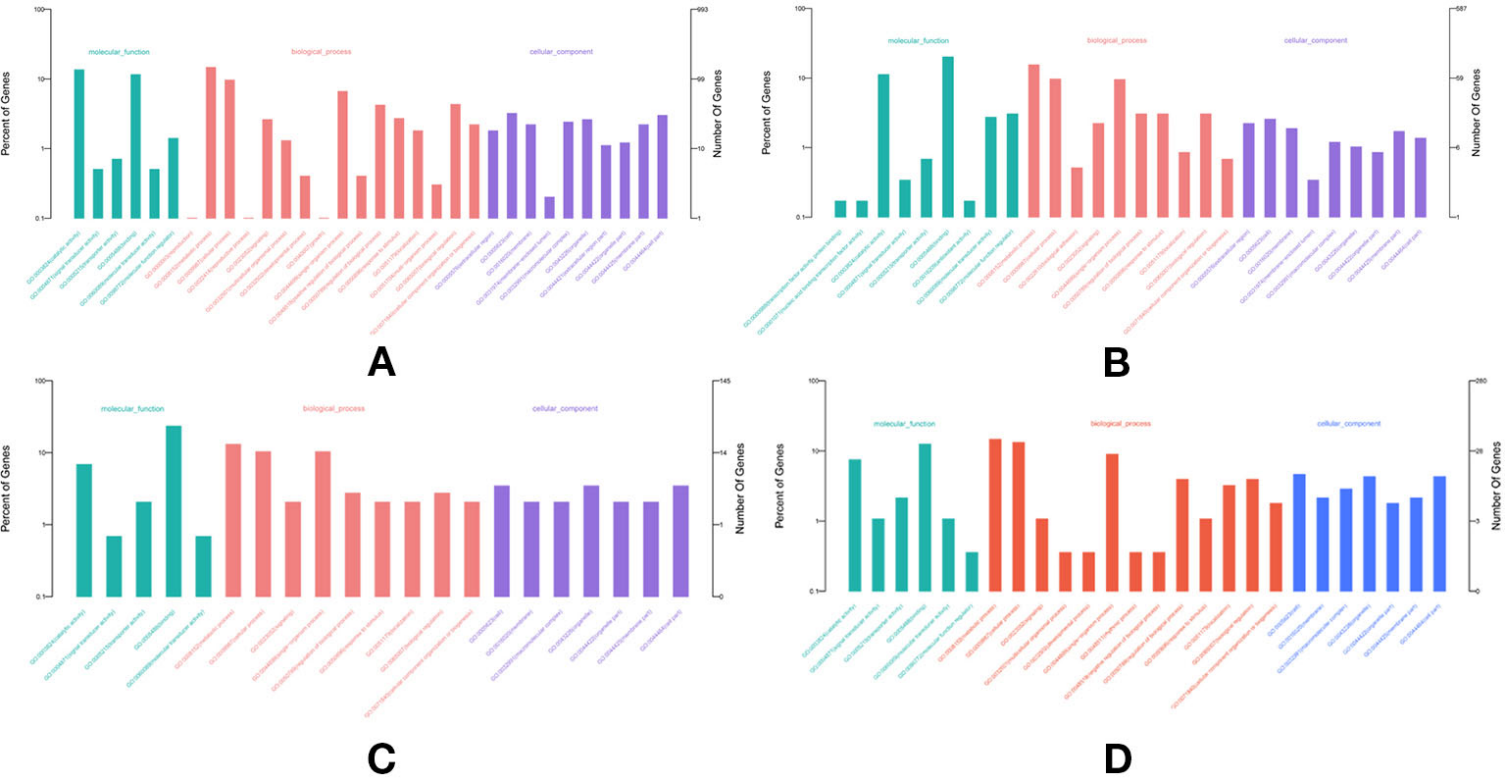
GO analysis results of new genes in the **(A)** KOC, **(B)** BSB, **(C)** RCC-L and **(D)** GF-L genomes.

In total, 682, 489, 691, and 1,356 new genes were predicted in the FL transcriptomes of GF-L, RCC-L, KOC, and BSB, respectively, and their gene annotations were analyzed with the Eukaryotic Orthologous Groups (KOG), KEGG, NR, Swiss-Prot, and Gene Ontology (GO) databases (**Table 2**). In the mapping of all new genes to terms in the GO database, 31/12/105/70, 99/70/403/304, and 68/30/247/163 genes could be grouped into the cellular component, molecular function and biological process categories in GF-L, RCC-L, KOC, and BSB, respectively. For GF-L, RCC-L, KOC, and BSB, within the cellular component category, the highest numbers of genes were assigned to the terms membrane (6, 19.35%), nucleus (3, 25%), integral components of membrane (18, 17.14%), and membrane (18, 25.71%), respectively; in the molecular function category, the highest numbers of genes were assigned to the terms protein binding (14, 14.14%), protein binding (11, 15.71%), protein binding (55, 13.65%), and protein binding (54, 17.76%), respectively; in the biological process categories, the highest numbers of genes were assigned to the terms DNA integration (12, 17.65%), oxidation-reduction process (8, 26.67%), proteolysis (72, 29.15%), and proteolysis (29, 17.79%), respectively (**Figure 2**). In the KEGG analysis, the genes in RCC-L and GF-L were mainly distributed in the pathways cellular processes, environmental information processing, genetic information processing, and organizational systems. RCC-L contained ten genes in the immune system pathway, while GF-L contained only three genes in this pathway. Transcripts containing complete CDSs, 5'-UTRs, and 3'-UTRs were defined as FL transcripts. In total, 2,689, 234, 144, and 245 FL transcripts were identified in GF-L, RCC-L, KOC, and BSB, respectively.

**Table 2:** New gene annotations.

Species	Database	KOG	KEGG	NR	Swiss-Prot	GO	Total unigenes	Total annotated
GF-L	Gene number	165	134	289	219	105	682	308
	Annotation ratio	24.19%	19.65%	42.38%	32.11%	15.40%	–	45.16%
RCC-L	Gene number	91	55	185	122	61	489	246
	Annotation ratio	18.61%	11.25%	37.83%	24.95%	12.47%	–	50.31%
KOC	Gene number	430	267	555	475	326	691	556
	Annotation ratio	62.23%	38.64%	80.32%	68.74%	47.18%	–	80.16%
BSB	Gene number	315	169	664	448	269	1356	667
	Annotation ratio	23.23%	12.46%	48.97%	33.04%	19.84%	–	49.19%

**Figure 3:**
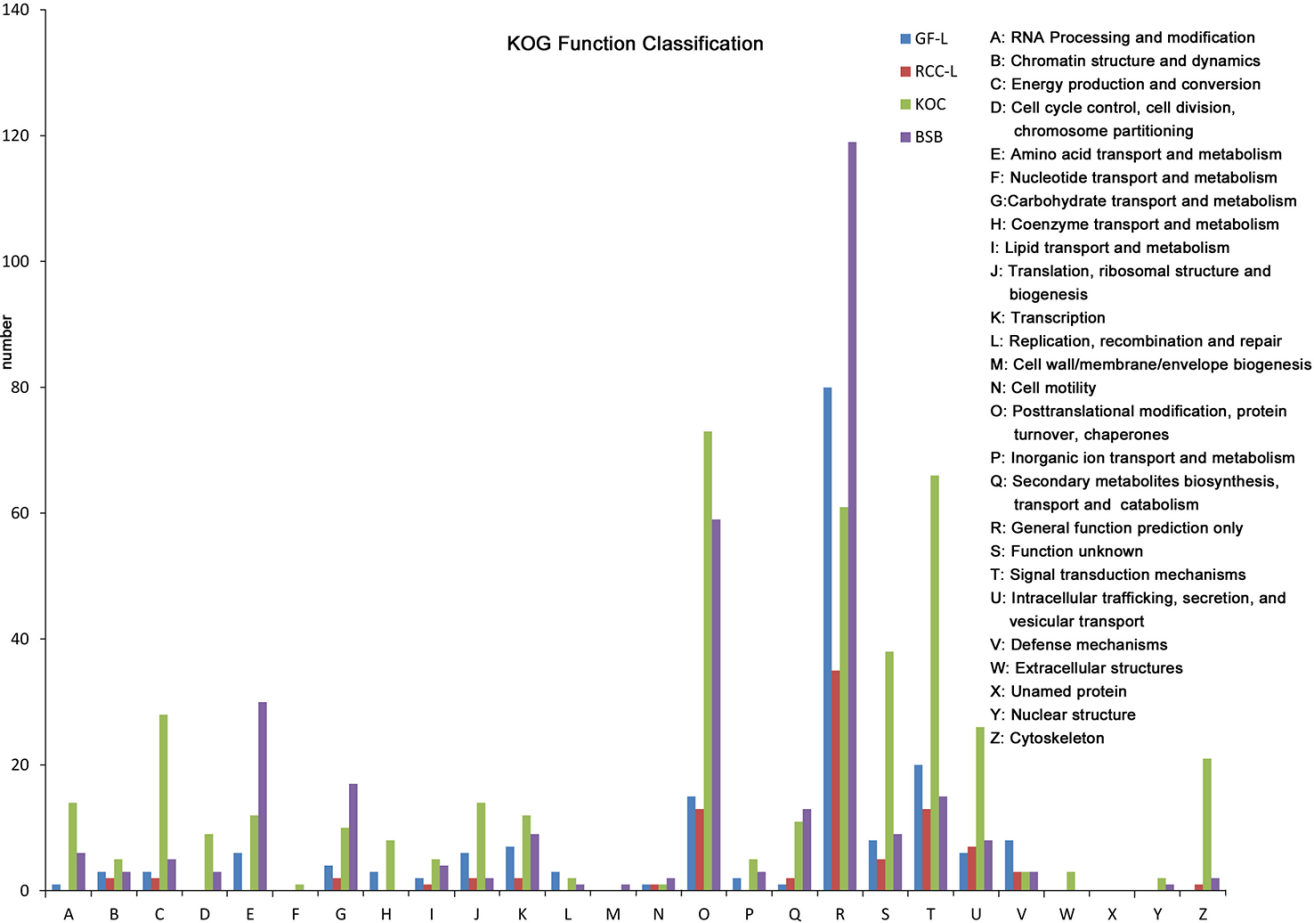
Analysis of Eukaryotic Orthologous Groups (KOG) functional classification of the new genes.

LncRNAs were an emerging topic in biology, and they have been shown to function as key regulators in a wide spectrum of biological processes. In this study, we identified 417, 531, 369, and 1,028 lncRNAs in GF-L, RCC-L, KOC, and BSB, respectively (**Supplementary Figure 1**) that provided candidate lncRNAs for further functional characterizations in crucian carp (**Figure 3**).

### Analysis of Fusion Genes

**Figure 4:**
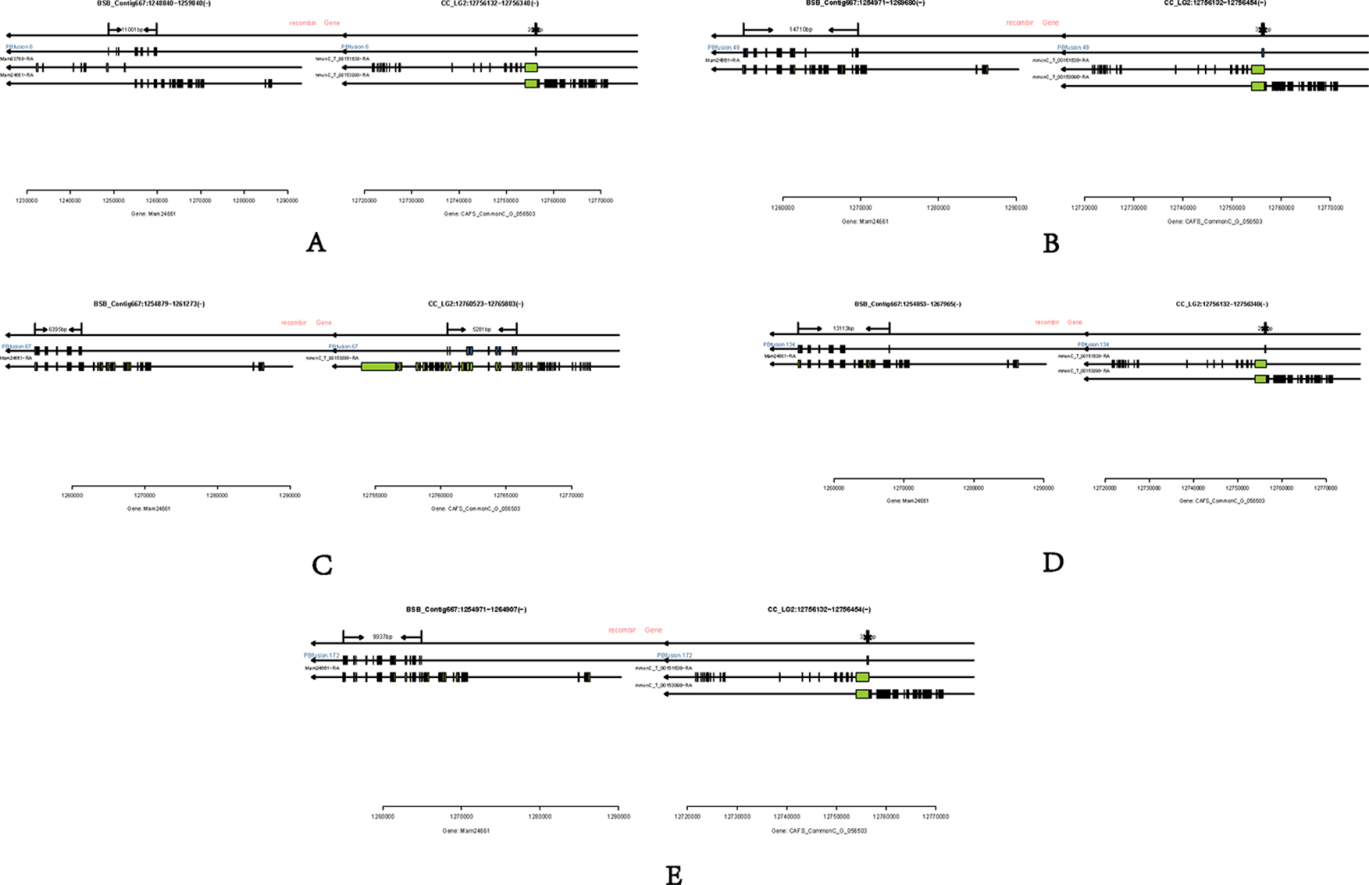
Fusion genes. **(A)** PBfusion6; **(B)** PBfusion49; **(C)** PBfusion67; **(D)** PBfusion134; **(E)** PBfusion172.

Long-read or FL cDNA sequences were useful for structural genomics studies such as the analysis of exon-intron structures. Using a stringent cut-off, we mapped the FL RCC-L and GF-L transcripts to the best-matched contig in the corresponding species and analyzed the possible exon-intron structures of the transcripts. Compared with the aligned CC and BSB genomes, in the FL transcriptome of RCC-L, there were 140 fusion genes including 140 reads. The analysis of the fusion genes showed that there were 52 genes from both the CC and RCC genomes, 5 genes from both the RCC and BSB genomes, and 5 genes from both the CC and BSB genomes. In the GF-L FL transcriptome, the analysis of the fusion genes showed that there were 272 genes, 35 of which were from both the CC and RCC genomes and 5 of which were from both the RCC and BSB genomes. This revealed that RCC-L and GF-L contained genetic material from KOC at the transcriptome level. The above results showed that most RCC-L and GF-L genetic materials were inherited from KOC (**Supplementary Table 2**). We described the structure of five genes from the CC and BSB genomes. These recombination genes (*PBfusion6*, *PBfusion49*, *PBfusion67*, *PBfusion134,* and *PBfusion172*) (**Figure 4**) were formed by recombination of the *Mam24661* gene from the CC genome and the *CAFS_CommonC_G_056503* gene from the BSB genome. The comparison of these recombination genes with the CC genome and the BSB genome revealed that the genes were more similar to the BSB genome than to the CC genome. For example, the percentage nucleotide identity values between *PBfusion6* and the CC genome and between *PBfusion6* and the BSB genome were 84.38% and 91.94%, respectively. Similarly, the percentage identity values between *PBfusion49*, *PBfusion67*, *PBfusion134,* and *PBfusion172* and the CC genome were 85.41%, 93.92%, 84.51%, and 84.51%, respectively. The percentage identities between *PBfusion49*, *PBfusion67*, *PBfusion134*, and *PBfuison172* and the BSB genome were 91.94%, 91.71%, 91.97%, and 92.47%, respectively (**Table 3**).

**Table 3:** The nucleotide identity percentages of fusion gene sequences in the RCC-L and GF-L genomes compared with the genomes of related species.

Fusion gene ID	Common carp genome		Blunt snout bream genome	
	Alignment location	Alignment Similarity (%)	Alignment location	Alignment similarity (%)
PB.fusion6	NC_031698.1 12756296-12756707	84.38	scaffold33: 2504726-2504911	91.94
PB.fusion49	NC_031698.112756298-12756707	85.41	scaffold33: 2504726-2504911	91.94
PB.fusion67	NC_031698.112756527-12756707	93.92	scaffold33: 2504726-2504906	91.71
PB.fusion134	NC_031698.112756298-12756707	84.51	scaffold33: 2504726-2504911	91.94
PB.fusion172	NC_031698.112756296-12756707	85.48	scaffold33: 2504726-2504911	92.47

### Orthologous Gene Analysis

The detailed third-generation RNA-seq data were shown in **Table 1**. In summary, 732 orthologous genes between GF-L, RCC-L, KOC, and BSB transcriptomes were recognized. The chimeric pattern was determined by distribution and variations of these orthologous genes. Eight gene patterns of the hybrid progeny were divided into three categories: chimaera, mutant, and biparental origin genes. The first included patterns 1–3, which had a single chimeric fragment consisting of successive alternating mutations from parent-specific variants, either with or without offspring-specific mutations. Pattern 1 and 2 contained genes with a single chimeric fragment composing of successive alternating mutations that came from parent-specific mutations. Pattern 3 included genes with chimeric fragments composing of successive alternating mutations from parent-specific mutations but with offspring-specific mutations. Chimeric genes of the first category taken up 48.2% and 46.5% of the genes from RCC-L and GF-L, respectively.

The second category contained chimeric genes with offspring-specific mutations. These genes were divided into patterns 4–6 formed by genes originating from both parents but with mutations distinct to the offspring. Chimeric genes from the second category contained 48.09% of genes in the overlapping mapped regions of RCC-L and 86.87% of that of GF-L.

**Figure 5:**
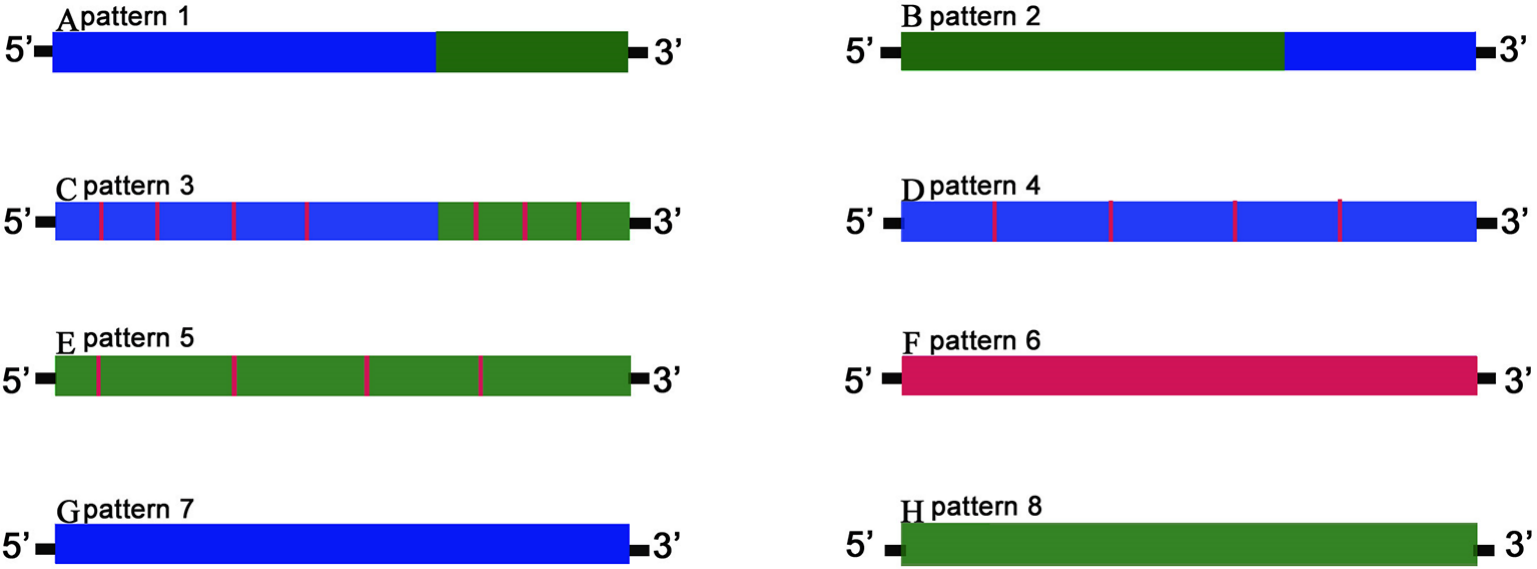
The gene chimeric model. Schematic diagrams of gene patterns for the offspring arising from the hybridization of KOC (K) and BSB (B). Blue bars marked K variation denote offspring fragments with the KOC-specific variants; green bars marked B variation show BSB-specific variants, and red bars marked F variation show offspring-specific variants. Genes were classified into three categories. The first category includes patterns 1–3 (**A–C,** respectively) in which chimeric genes had single or multiple chimeric fragments consisting of successive alternating variations from parent-specific variants, either with or without offspring-specific mutations. The second category includes patterns 4–6 (**D–F**, respectively), consisting of genes derived from either or both progenitors but with mutations unique to the offspring. The third category includes patterns 7–8 (**G–H**, respectively), in which genes are derived exclusively from one parent.

Finally, the third category contained pattern 7 and pattern 8, in which of genes originated only from one parent. Pattern 7 contained genes from the KOC parent, while pattern 8 contained genes from the BSB parent. The third category included 3.70% of genes in the overlapping mapped regions of RCC-L and 8.30% of that of GF-L (**Figure 5**).

### Evaluation of Ka/Ks Ratios in Inferred Orthologous Gene Pairs Between GF-L and KOC

**Figure 6:**
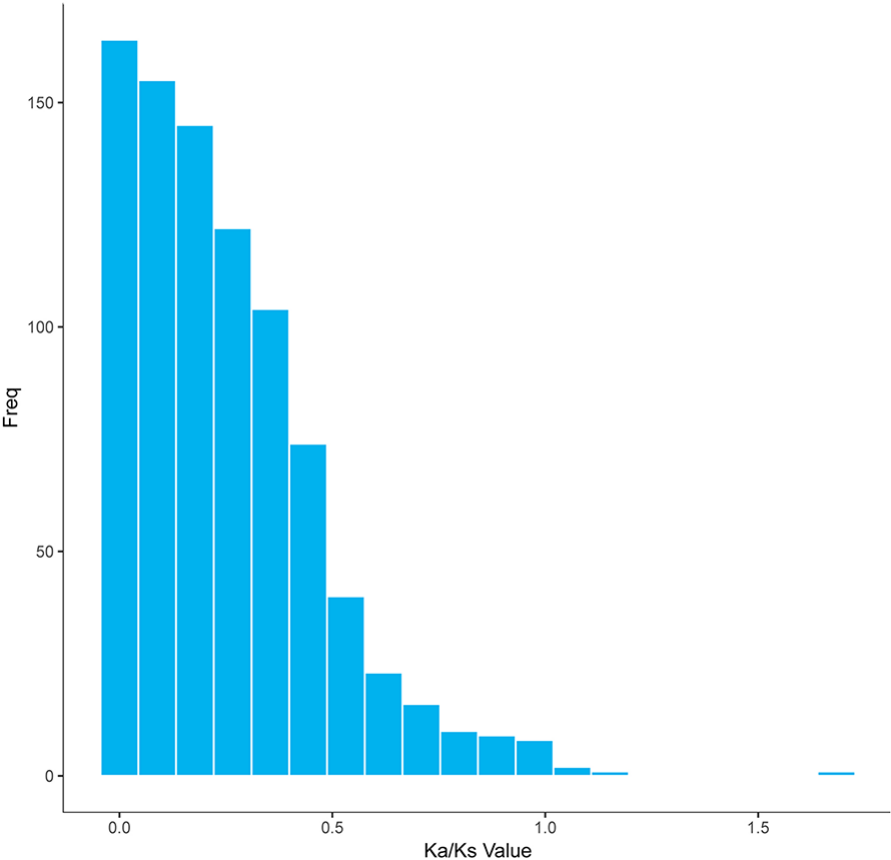
Distribution of the Ka/Ks ratios in 5,621 putative orthologous gene pairs between GF-L and KOC.

In a long-term breeding experiment, we found that the GF originated from the distant hybridization of KOC (♀) and BSB (♂) (Wang et al., 2018). We believed that the GF was derived *via* the route KOC–RCC–GF. To search for candidate genes involved in the evolution or domestication of GF, we investigated the selective pattern of genes between GF-L and KOC. We compared the FL transcript data that included 59,274 and 27,516 transcripts, removed those transcripts with multiple possible orthologs, and finally confirmed 5,621 inferred orthologous gene pairs between GF-L and KOC. Afterwards, the Ka and Ks rates and the Ka/Ks ratio of each gene pair were calculated. As shown in **Figure 6**, the Ka/Ks values of most gene pairs were less than one, and only six gene pairs exhibited Ka/Ks values greater than one. These data showed that most genes were under the process of purifying selection during the evolution or domestication of GF. The role of those genes under strong positive selection process must be further studied (**Figure 6**).

### The Alignment of the Resequenced RCC-L and GF-L Genomes With the Genomes of Related Species

The resequenced RCC-L and GF-L genomes were mapped to the corresponding sequences in the CC, BSB and RCC genomes as references. The results were shown in **Table 4**. The sequence similarities of the resequenced RCC-L and GF-L genomes to the CC genome were 92.52% and 90.18%, respectively. However, the sequence similarities of the resequenced RCC-L and GF-L genomes to the BSB genome were 57.33% and 49.18%, respectively. Because KOC was a variety of CC, we concluded that the similarity of RCC-L to KOC was higher than that of RCC-L to BSB. Regarding the RCC-L and RCC genomes, the nucleotide similarities of RCC-L and GF-L to the RCC genome were 99.66% and 99.28%, respectively, showing genomic DNA variation in the RCC-L and GF-L genomes (**Table 4**).

**Table 4:** The resequenced RCC-L and GF-L genomes compared with the genomes of related species.

Species	Common carp genome		Blunt snout bream genome		Red crucian carp genome	
	The number of alignment reads	Alignment similarity (%)	The number of alignment reads	Alignment similarity (%)	The number of alignment reads	Alignment similarity (%)
RCC-L	694868799	92.52%	437080629	57.33%	761625845	99.66%
GF-L	366968429	90.18%	230436483	49.18%	376491759	99.28%

### Identification of SNPs and SVs in the Newly Established RCC-L and GF-L Genomes

After resequencing the RCC-L and GF-L genomes, we obtained 135.94 G and 97.79 G clean reads, respectively. Then, we evaluated SNPs and recognized 29,376,810 SNPs between the sequences of RCC-L and the CC and 39,260,783 SNPs between the sequences of GF-L and the CC. SNPs were classified as either transitions or transversions. For RCC-L, we identified 2,148,286 SNPs in CDSs, of which 1,145,605 and 1,002,681 coding SNPs were categorized as non-synonymous and synonymous nucleotide substitutions, respectively (**Table 5**). For GF-L, we identified 2,653,293 SNPs in CDSs, of which 1,436,045 and 1,217,248 coding SNPs were categorized as Ka and Ks nucleotide substitutions, respectively (**Table 5**).

**Table 5:** Summary of SNPs in the RCC-L and GF-L genomes.

Item	RCC-L		GF-L	
	Mapping to CC	Ratio	Mapping to CC	Ratio
SNP number	29376810	1	39260783	1
CDS	2148286	0.073128634	2653293	0.067581255
Synonymous	1002681	0.034131718	1217248	0.03100417
Missense	754025	0.025667355	937279	0.023873161
Stopgain	7076	0.00024087	9772	0.0002489
Stoploss	1241	4.22442E-05	1709	4.35294E-05
Other 5'UTR 3'UTR	383263 313363 690404	0.013046447 0.010667019 0.023501667	487285 375173 810500	0.012411495 0.009555923 0.02064401
Intronic Splicing	10300931 230239	0.350648386 0.00783744	13299414 280064	0.338745511 0.007133429
Upstream	4332007	0.147463492	5909459	0.150518114
Downstream	3216301	0.109484352	4383919	0.111661527
Intergenic	8145279	0.277269009	11548961	0.294160231

SV analysis of the RCC-L genome indicated that five types of SVs were detected, with 275,923 DELs, 421,191 CTXs, 28,008 DUPs, 896 INVs, and 426,625 intra-chromosomal translocations (ITXs) identified by mapping to the CC genome.

Five types of SVs that were 205,795 DELs, 282,666 CTXs, 20,667 DUPs, 743 INVs, and 4,078 ITXs were detected by the approach of SV mapping of the GF-L genome.

## Discussion

### The Significance of Hybridization

Genome hybridization was an important evolutionary event that produced species with novel capabilities (Sriswasdi et al., 2019). This process combined the genetic material from parental species into hybrid offspring, resulting in changes in gene regulation and expression in the hybrid progeny (Mallet, 2007; Stelkens and Seehausen, 2009; Liu, 2010). Thus, hybrids could exhibit many advantageous traits, such as rapid growth, strong disease resistance, and strong environmental tolerance (Bartley et al., 2000; Liu et al., 2016). In previous research (Wang et al., 2018), the homodiploid hybrids (RCC-L and GF-L) originated from the distant hybridization of KOC (♀) × BSB (♂) exhibited significant changes in shape. These RCC-L and GF-L had bright black eyes and a white base colour covered with red and yellow patterns (**Figure 1**). The results provided clear evidence for the pathway of the formation of the GF: KOC (KOC as a variety of CC)-RCC-GF (**Figure 1**). GF-L and RCC-L were shown to be homodiploids mainly originated from the genome of KOC but with some DNA fragments from the BSB genome (**Figures 4** and **5**).

Transcriptome profiling *via* RNA sequencing revealed large quantities of high-quality transcripts produced for GF-L, RCC-L, KOC, and BSB (**Table 1**). Transcriptome annotation revealed that the numbers of new genes in the RCC-L and GF-L genomes were similar to that in the KOC genome; however, the numbers of new genes in the RCC-L and GF-L genomes were significantly lower than that in the BSB genome (P < 0.05) (**Table 2**). The results showed that GF-L and RCC-L contained the genetic materials of KOC and BSB.

In terms of fusion genes, GF-L and RCC-L showed different patterns. RCC-L had five fusion genes from the CC and BSB genomes. However, GF-L had some fusion genes from the CC genome with BSB DNA fragments. The presence of fusion genes, which was probably due to the gene conversion from the parental genome, showed further evidence for DNA variation in GF-L and RCC-L (**Figures 3** and **4**).

The comparison of the orthologous genes provided a rich and detailed resource for researchers to explore the relationships between hybrid traits and chimeric genes (**Figure 5**).

The Ka/Ks results revealed that 868 genes were under negative selection process (Ka/Ks < 1) in GF-L; however, only eight genes were under positive selection process (Ka/Ks > 1). These results showed that GF-L had rapidly evolved. Of these genes, those with non-synonymous substitutions may have contributed to accelerating the evolution of GF-L (**Table 5**, **Figure 6**).

The results of mapping the sequences from the genome resequencing of RCC-L and GF-L to each of the CC and BSB genomes provided further evidence that RCC-L and GF-L derived from the hybridization of both KOC and BSB. The genome of KOC was consistently the same as that of CC. The average similarity of both the GF-L and RCC-L genomes to the CC genome was significantly higher than to the BSB genome, further supported the hypothesis that the RCC-L and GF-L genomes were mainly inherited from KOC but with some DNA fragments from BSB (**Table 4**).

The large number of non-synonymous substitutions and indels suggested significant changes in the genomic structure, clearly reflecting instability in the newly established homodiploid lineages (RCC-L and GF-L) and helping to accelerate the formation of new species.

### The Significance of Chimeric Genes in Hybrid Fish

The chimeric genes were mainly hybrid genes formed by splicing gene sequences with different sources and functions, mainly for the insertion of a parental gene (point insertion and fragment insertion), a point mutation fragment of a hybrid progeny gene sequence, or the deletion of one of the parents' sequences (Flagel and Wendel, 2009).

The formation of chimeric genes not only enriched the diversity and complexity of transcriptomes and proteomes but also had very important biological functions. In the process of distant hybridization in fish, a large number of chimeric genes and mutant genes were enriched in functional and signalling pathways such as metabolism, and development. For example, previous studies found that 9.67%–11.06% of the genes found in allotetraploids belonged to the chimeric genes derived from female RCC and male CC, some of which (*CSNK*, *CDC*, *RAD,* and *UBE*) were involved in cell cycle regulation or were associated with functions such as DNA damage repair by recombination (Liu et al., 2016). Similarly, we found that 19.04% of the chimeric genes were present in the distant hybrids of white crucian carp (WCC) and RCC, and these chimeric genes were significantly associated with the dominant traits of the hybrids. The hybrids had the characteristics of fast growth, strong resistance, and being rich in amino acids (Liu et al., 2018). The GF-L was the offspring of self-mating RCC-L. RCC-L was formed by the hybridization of KOC and BSB. Through the alignment of orthologous genes, it was found that the RCC-L and GF-L genomes contained 48.20% and 46.50% chimeric genes, respectively. The presence of these chimeric genes had a significant impact to some extent on RCC-L and GF-L features. For example, the female parent KOC had two pairs of barbels, but the RCC-L and GF-L had not barbels and were more similar to crucian carp than to KOC morphologically. As a new type of fish, GF-L had a high fertilization rate (91.62%) and a hatching rate (82.67%). GF-L presented very beautiful phenotypes, such as twin tails, blue eyes and a white body accompanied by red spots. These phenotypes were very different from any other GF phenotype, showed that the GF-L lineage had great potential in the ornamental market. Compared with a GF, a female GF-L could produce tens of thousands of eggs and a male GF-L could produce 1–2 ml of semen, which was important factors in increasing the production of GF. A large number of new type of GF with high ornamental value can be obtained by crossing GF-L with ordinary GF. In summary, the formation of GF-L was very important to both evolutionary biology and fish genetic breeding.

## Data Availability Statement

The PacBio SMRT reads in this study have been submitted to the SRA database of the National Center (accession numbers: SRR8791238, SRR8793544, SRR8791143, SRR8791174, SRR10586222, and SRR10589322).

## Ethics Statement

The animal study was reviewed and approved by the Institute of Experimental Animals, Hunan Province, China.

## Author Contributions

This study was conceived and designed by SL. YW and HT contributed to the experimental work, most statistical analysis and writing the manuscript. RZ, QQ, JW, SW, and MZ designed the primers and performed the bioinformatics analysis. YW contributed to the experimental materials collection. MT, CZ, MM, and BC captured photographs. All authors read and approve the final manuscript.

## Funding

This work was supported by the National Natural Science Foundation of China (Grant Nos. 31430088, 31730098), the earmarked fund for China Agriculture Research System (Grant No. CARS-45), the Hunan Provincial Natural Science and Technology Major Project (Grant No. 2017NK1031), the Cooperative Innovation Center of Engineering, High-level Talent Agglomeration Program of Hunan, China (2019RS1044) and New Products for Developmental Biology of Hunan Province (Grant No. 20134486).

## Conflict of Interest

The authors declare that the research was conducted in the absence of any commercial or financial relationships that could be construed as a potential conflict of interest.
