# Supplementary material for: The Hybrid Genome of a New Goldfish-Like Fish Lineage Provides Insights Into the Origin of the Goldfish
Source: Front Genet. 2020 Mar 3;11:122. doi: 10.3389/fgene.2020.00122 (PMC7063666; doi:10.3389/fgene.2020.00122)
Supplement: Supplementary file 1 [file DataSheet_1.docx]

**Supplementary Table**

Supplementary Table 1. 16-mer barcode sequences used for PacBio barcoding libraries

| Species name | sequence |
| --- | --- |
| Koi carp | ACAGTCTATACTGCTG |
| Blunt snout bream | TGTGTATCAGTACATG |
| Red crucial carp-like homodiploid fish | GATCTCTACTATATGC |
| Goldfish-like homodiploid fish | TACTAGAGTAGCACTC |
|  |  |

Supplementary Table 2. The Fusion gene structure analysis

| Fusion gene | Gene structure location |
| --- | --- |
| RCC-L_PBfusion.4 | CC_000000774:1275252-1370192(+)+RCC_Scaffold3373:53620-54109(+) |
| RCC-L_PBfusion.5 | CC_LG40:19815352-19815934(+)+RCC_Scaffold93:1230407-1240999(+) |
| RCC-L_PBfusion.6 | BSB_Contig667:1248840-1259840(-)+CC_LG2:12756132-12756340(-) |
| RCC-L_PBfusion.8 | CC_000000719:671916-672421(-)+RCC_Scaffold47:2307281-2316668(-) |
| RCC-L_PBfusion.12 | CC_LG28:12091272-12101905(-)+RCC_Scaffold794:34125-35760(-) |
| RCC-L_PBfusion.14 | CC_LG13:11803001-11803206(+)+RCC_Scaffold30:8964410-8968948(+) |
| RCC-L_PBfusion.15 | CC_000000158:106573-109696(+)+RCC_Scaffold380:1249656-1249864(+) |
| RCC-L_PBfusion.18 | CC_000001032:52970-149330(+)+RCC_Scaffold1734:268955-269049(+) |
| RCC-L_PBfusion.24 | BSB_Contig400:621288-625934(+)+RCC_Scaffold501:1372098-1450750(+) |
| RCC-L_PBfusion.27 | CC_000000135:283418-283845(+)+RCC_Scaffold141:906329-907057(+) |
| RCC-L_PBfusion.28 | CC_000028846:656616-661195(-)+RCC_Scaffold374:208032-219233(-) |
| RCC-L_PBfusion.30 | CC_000028946:1253347-1253599(+)+RCC_Scaffold58:4170837-4171889(+) |
| RCC-L_PBfusion.32 | CC_000000072:176940-178771(+)+RCC_Scaffold1996:18022-19853(-) |
| RCC-L_PBfusion.33 | CC_000002477:309442-336509(-)+RCC_Scaffold3032:92731-98529(-) |
| RCC-L_PBfusion.36 | CC_LG4:867553-867828(+)+RCC_Scaffold220:129015-131554(+) |
| RCC-L_PBfusion.39 | CC_LG31:17369125-17369691(+)+RCC_Scaffold2087:713232-731370(+) |
| RCC-L_PBfusion.41 | CC_000010593:74859-75224(-)+RCC_Scaffold366:733122-738325(-) |
| RCC-L_PBfusion.46 | CC_LG26:7618449-7619335(-)+RCC_Scaffold3709:158156-161575(-) |
| RCC-L_PBfusion.49 | BSB_Contig667:1254971-1269680(-)+CC_LG2:12756132-12756454(-) |
| RCC-L_PBfusion.55 | CC_000002881:26480-26851(+)+RCC_Scaffold351:208591-211615(+) |
| RCC-L_PBfusion.61 | CC_000028946:1267178-1267577(+)+RCC_Scaffold1061:60003-62347(+) |
| RCC-L_PBfusion.63 | CC_000010593:74860-75471(+)+RCC_Scaffold366:733401-739514(-) |
| RCC-L_PBfusion.64 | CC_LG14:9963245-9963433(-)+RCC_Scaffold1892:603504-605032(-) |
| RCC-L_PBfusion.67 | BSB_Contig667:1254879-1261273(-)+CC_LG2:12760523-12765803(-) |
| RCC-L_PBfusion.69 | CC_000001383:406205-406377(-)+RCC_Scaffold4199:150049-159918(-) |
| RCC-L_PBfusion.75 | CC_000010593:74858-75472(-)+RCC_Scaffold366:733401-739514(-) |
| RCC-L_PBfusion.84 | CC_000028871:1095729-1111650(+)+RCC_Scaffold5:2380362-2381160(+) |
| RCC-L_PBfusion.85 | BSB_Contig1098:2137675-2139729(-)+RCC_Scaffold238:2940754-2946882(-) |
| RCC-L_PBfusion.90 | CC_LG16:1200554-1224800(+)+RCC_Scaffold782:346786-377921(+) |
| RCC-L_PBfusion.95 | CC_LG45:11662059-11688613(+)+RCC_Scaffold1852:7681-32381(+) |
| RCC-L_PBfusion.99 | CC_000028946:1267178-1267577(+)+RCC_Scaffold45:5948322-5948891(+) |
| RCC-L_PBfusion.101 | CC_LG35:4015215-4017747(+)+RCC_Scaffold49:2809890-2811922(+) |
| RCC-L_PBfusion.102 | CC_000028946:1253347-1253599(+)+RCC_Scaffold26:4149235-4158194(+) |
| RCC-L_PBfusion.105 | CC_LG42:4983789-4983982(+)+RCC_Scaffold15:4582388-4583417(+) |
| RCC-L_PBfusion.122 | BSB_Contig947:795976-802546(-)+RCC_Scaffold178:1029963-1048551(-) |
| RCC-L_PBfusion.123 | CC_LG34:8114081-8114245(-)+RCC_Scaffold96:3723500-3724790(-) |
| RCC-L_PBfusion.125 | CC_000010593:74859-75224(-)+RCC_Scaffold366:733123-738325(-) |
| RCC-L_PBfusion.127 | CC_LG45:3052984-3054025(+)+RCC_Scaffold69:3248016-3248058(+) |
| RCC-L_PBfusion.128 | CC_000000701:19158-22180(-)+RCC_Scaffold361:1113124-1117549(-) |
| RCC-L_PBfusion.131 | CC_000028946:1606404-1606805(+)+RCC_Scaffold178:2695995-2707890(+) |
| RCC-L_PBfusion.132 | CC_000010593:74859-75224(-)+RCC_Scaffold366:733070-738798(-) |
| RCC-L_PBfusion.134 | BSB_Contig667:1254853-1267965(-)+CC_LG2:12756132-12756340(-) |
| RCC-L_PBfusion.135 | CC_000010593:74859-75224(-)+RCC_Scaffold366:733123-738334(-) |
| RCC-L_PBfusion.140 | CC_000010593:74859-75224(-)+RCC_Scaffold366:733083-739514(-) |
| RCC-L_PBfusion.141 | CC_LG6:36122-114779(-)+RCC_Scaffold90:447399-459388(-) |
| RCC-L_PBfusion.142 | CC_LG33:15384624-15385778(+)+RCC_Scaffold2344:1140615-1145806(+) |
| RCC-L_PBfusion.143 | CC_000000712:1086200-1141687(+)+RCC_Scaffold782:43175-47293(+) |
| RCC-L_PBfusion.147 | CC_000029006:145437-145952(-)+RCC_Scaffold154:2027221-2030021(-) |
| RCC-L_PBfusion.148 | CC_000000719:1511024-1512582(-)+RCC_Scaffold421:891211-930582(-) |
| RCC-L_PBfusion.149 | CC_000028946:1267308-1267551(+)+RCC_Scaffold53:5345594-5345717(+) |
| RCC-L_PBfusion.158 | CC_LG38:16562832-16564101(-)+RCC_Scaffold366:734132-738326(-) |
| RCC-L_PBfusion.164 | CC_LG42:2517204-2517396(-)+RCC_Scaffold15:5930589-5934962(-) |
| RCC-L_PBfusion.172 | BSB_Contig667:1254971-1264907(-)+CC_LG2:12756132-12756454(-) |
| RCC-L_PBfusion.173 | CC_LG18:8807791-8810572(-)+RCC_Scaffold7:2685215-2685376(-) |
| RCC-L_PBfusion.174 | CC_LG9:2731948-2737302(-)+RCC_Scaffold61:2854062-2854669(-) |
| RCC-L_PBfusion.175 | BSB_Contig223:3297727-3303723(+)+RCC_Scaffold327:522921-543160(+) |
| RCC-L_PBfusion.180 | CC_000000591:591862-593898(-)+RCC_Scaffold48:9005708-9007104(-) |
| RCC-L_PBfusion.184 | BSB_Contig275:5216-31059(+)+RCC_Scaffold1734:298969-299242(+) |
| RCC-L_PBfusion.189 | CC_LG7:483281-487649(+)+RCC_Scaffold144:778701-779007(+) |
| RCC-L_PBfusion.191 | CC_000001383:406205-406377(-)+RCC_Scaffold4199:159731-166681(-) |
| RCC-L_PBfusion.194 | CC_LG14:9989353-9989621(+)+RCC_Scaffold144:753661-797402(+) |
| RCC-L_PBfusion.204 | CC_000028810:115299-116031(-)+RCC_Scaffold1508:1182987-1185973(-) |
| GF-L_PBfusion.214 | CC_LG38:16651618-16651717(-)+RCC_Scaffold58:4170279-4171891(+) |
| GF-L_PBfusion.202 | CC_000028946:1279604-1279998(+)+RCC_Scaffold186:1132795-1135015(-) |
| GF-L_PBfusion.17 | CC_000028946:1267343-1267520(+)+RCC_Scaffold5349:1455659-1460167(-) |
| GF-L_PBfusion.2 | CC_000028946:1279262-1442211(+)+RCC_Scaffold298:105203-105877(-) |
| GF-L_PBfusion.3 | CC_000028946:1267281-1267554(+)+RCC_Scaffold1402:1210416-1215577(+) |
| GF-L_PBfusion.215 | CC_000028946:1253346-1253576(+)+RCC_Scaffold4521:12057-13063(-) |
| GF-L_PBfusion.211 | CC_000028946:1267224-1267581(+)+RCC_Scaffold63:3324542-3332131(-) |
| GF-L_PBfusion.87 | CC_000028946:1253329-1253489(+)+RCC_Scaffold4521:12057-13605(-) |
| GF-L_PBfusion.84 | CC_LG34:13673779-13702916(+)+RCC_Scaffold1930:6119-6299(-) |
| GF-L_PBfusion.83 | CC_LG31:1047045-1047131(+)+RCC_Scaffold140:3072911-3075836(+) |
| GF-L_PBfusion.82 | CC_000028946:1267151-1267548(+)+RCC_Scaffold507:210185-218509(-) |
| GF-L_PBfusion.117 | CC_LG6:18480566-18485201(+)+RCC_Scaffold4357:72967-73448(-) |
| GF-L_PBfusion.261 | CC_000001295:5986-6129(+)+RCC_Scaffold96:160742-171404(-) |
| GF-L_PBfusion.198 | CC_000028849:1599491-1599739(+)+RCC_Scaffold3026:1089551-1098862(+) |
| GF-L_PBfusion.97 | CC_000029029:2900288-2900390(+)+RCC_Scaffold499:1632-5599(+) |
| GF-L_PBfusion.93 | CC_000028905:798273-798361(-)+RCC_Scaffold246:364766-427204(-) |
| GF-L_PBfusion.37 | CC_000010593:74871-75175(-)+RCC_Scaffold366:733092-739514(-) |
| GF-L_PBfusion.101 | CC_000028946:1267280-1267582(+)+RCC_Scaffold13:7038660-7039632(+) |
| GF-L_PBfusion.109 | CC_000028946:1267159-1267586(+)+RCC_Scaffold134:335767-337558(+) |
| GF-L_PBfusion.271 | CC_LG32:32626-33386(-)+RCC_Scaffold3571:264357-271447(+) |
| GF-L_PBfusion.187 | CC_000028946:1253346-1253560(+)+RCC_Scaffold186:1114444-1115901(-) |
| GF-L_PBfusion.42 | CC_LG16:1200770-1224800(+)+RCC_Scaffold782:376336-377921(+) |
| GF-L_PBfusion.45 | CC_000028946:1253346-1253534(+)+RCC_Scaffold3361:44200-48869(+) |
| GF-L_PBfusion.174 | CC_000028946:1256667-1256816(+)+RCC_Scaffold58:4170369-4171885(+) |
| GF-L_PBfusion.53 | CC_000028946:1267280-1267580(+)+RCC_Scaffold186:1051286-1057349(-) |
| GF-L_PBfusion.54 | CC_000028946:1267159-1267325(+)+RCC_Scaffold400:682850-684471(+) |
| GF-L_PBfusion.57 | CC_LG31:7405551-7406688(+)+RCC_Scaffold67:7422728-7424929(+) |
| GF-L_PBfusion.259 | BSB_Contig836:253112-253193(+)+RCC_Scaffold413:1854319-1858361(+) |
| GF-L_PBfusion.251 | BSB_Contig527:205820-205940(+)+RCC_Scaffold5337:18056-20891(+) |
| GF-L_PBfusion.255 | BSB_Contig336:1991684-1991720(+)+RCC_Scaffold310:1375425-1378180(+) |
| GF-L_PBfusion.257 | CC_000028946:1269641-1269936(+)+RCC_Scaffold1:329613-330277(-) |
| GF-L_PBfusion.167 | CC_000028946:1268069-1268197(+)+RCC_Scaffold4479:395616-397263(+) |
| GF-L_PBfusion.66 | CC_000028946:1590291-1590549(+)+RCC_Scaffold171:776679-779161(-) |
| GF-L_PBfusion.63 | CC_LG33:11712294-11734787(-)+RCC_Scaffold390:451072-453470(+) |
| GF-L_PBfusion.152 | CC_000001186:80525-80570(-)+RCC_Scaffold122:3235896-3236810(-) |
| GF-L_PBfusion.70 | BSB_Contig113:464683-465053(-)+RCC_Scaffold286:163534-164526(-) |
| GF-L_PBfusion.237 | CC_000004685:3604-26974(+)+RCC_Scaffold51:5179000-5184117(+) |
| GF-L_PBfusion.236 | CC_LG4:9638675-9638727(+)+RCC_Scaffold114:143826-151968(+) |
| GF-L_PBfusion.232 | CC_000000982:71470-164903(-)+RCC_Scaffold82:3354846-3358763(+) |

**Supplementary Figure**

**Supplementary Figure 1 Length distribution of lncRNA**


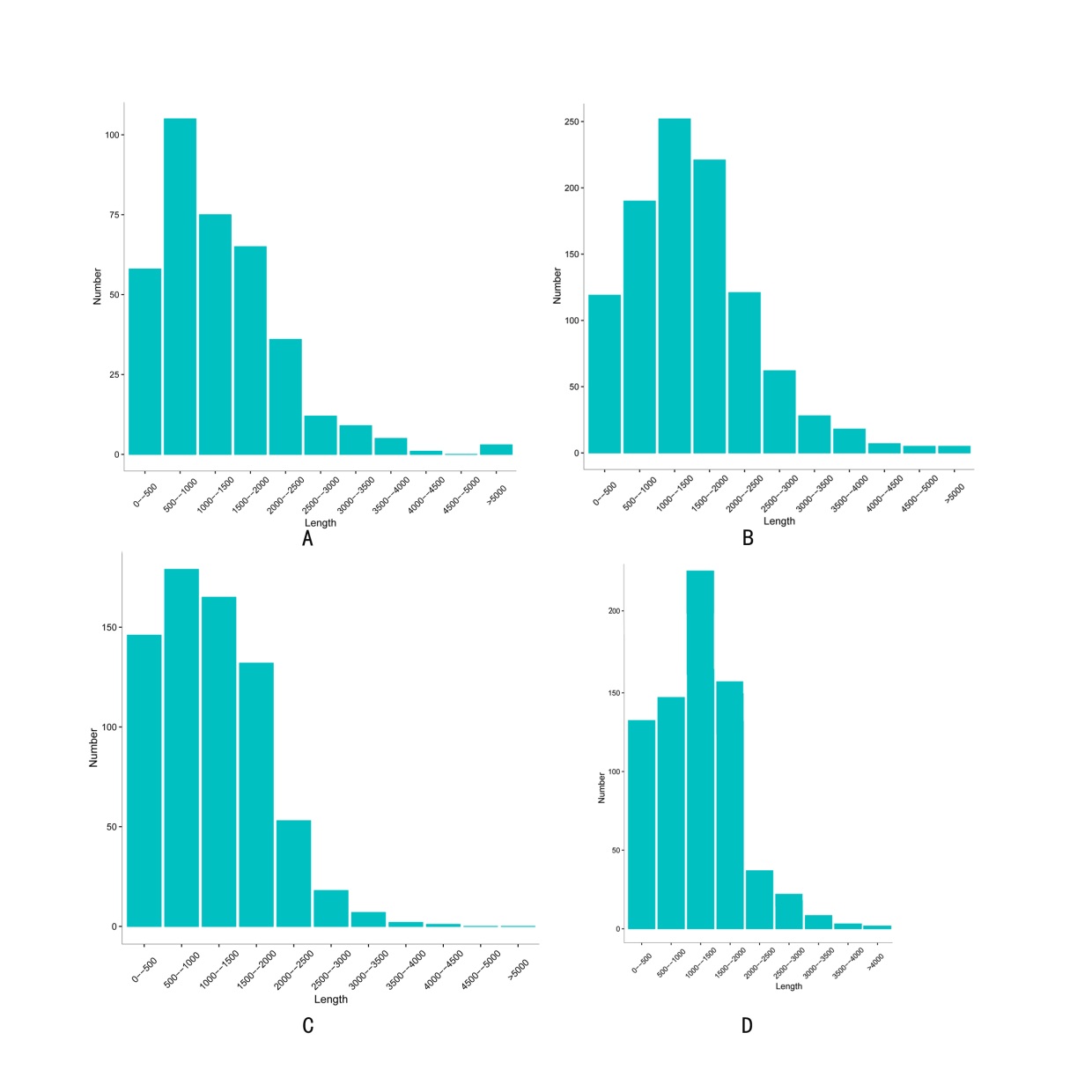


**A: KOC; B: BSB; C: RCC-L; D: GF-L**
